# Utility of Tear Osmolarity Measurement in Diagnosis of Dry Eye Disease

**DOI:** 10.1038/s41598-020-62583-x

**Published:** 2020-03-26

**Authors:** Bezhod Tashbayev, Tor Paaske Utheim, Øygunn Aass Utheim, Sten Ræder, Janicke Liaaen Jensen, Mazyar Yazdani, Neil Lagali, Valeria Vitelli, Darlene A. Dartt, Xiangjun Chen

**Affiliations:** 1 Department of Oral Surgery and Oral Medicine, Faculty of Dentistry, University of Oslo, Oslo, Norway; 2 The Norwegian Dry Eye Clinic, 0366 Oslo, Norway; 3 Department of Medical Biochemistry, Oslo University Hospital, Ullevål, 0450 Oslo Norway; 4 Department of Ophthalmology, Oslo University Hospital, 0450 Oslo, Norway; 5 Institute of Oral Biology, Faculty of Dentistry, University of Oslo, 0318 Oslo, Norway; 6 Department of Ophthalmology, Vestre Viken Hospital Trust, 3019 Drammen, Norway; 7 Department of Ophthalmology, Sørlandet Hospital Arendal, 4604 Arendal, Norway; 8 National Centre for Optics, Vision and Eye Care, Faculty of Health Sciences, University of South Eastern Norway, 3603 Kongsberg, Norway; 9 Department of Clinical and Experimental Medicine, Faculty of Health Sciences, Linköping University, Linköping, Sweden; 10 Oslo Center for Biostatistics and Epidemiology, Department of Biostatistics, University of Oslo, Oslo, Norway; 11 Schepens Eye Research Institute/Massachusetts Eye and Ear, Department of Ophthalmology, Harvard Medical School, Boston, MA USA

**Keywords:** Conjunctival diseases, Diagnostic markers

## Abstract

The prevalence of dry eye disease is high worldwide and poses a great burden on patients’ daily lives. Accurate diagnosis of the disease is important, and it requires application of various methods. Hyperosmolarity is believed to be the disease marker and thus measuring it provides useful information. In this study we investigated utility of tear osmolarity measured with TearLab osmometer, along with other diagnostic tests (Ocular Surface Disease Index questionnaire, Tear film break-up time, Ocular Protection Index, Ocular Surface Staining, Schirmer I test, Meibomian gland functionality in 757 patients (1514 eyes) with dry eye disease and 29 healthy controls (58 eyes). Statistical differences between the patient group and the control group were observed for all the tests apart from tear osmolarity, regardless of cut-off value (>308 mOsm/L, >316 mOsm/L, and inter-eye difference >8 mOsm/L). Moreover, in the receiver operating characteristics curve analyses tear osmolarity measurement could not discriminate dry eye disease pathological scores. Therefore, our study suggests that tear osmolarity measured with TearLab osmometer cannot be used as a key indicator of DED.

## Introduction

The International Dry Eye Workshop II 2017 defines dry eye disease (DED) as “… *a multifactorial disease of the ocular surface characterized by a loss of homeostasis of the tear film, and accompanied by ocular symptoms, in which tear film instability and hyperosmolarity, ocular surface inflammation and damage, and neurosensory abnormalities play etiological roles*”^1^. The symptoms of DED include ocular burning, foreign body sensation, soreness, stinging, irritation, reduced visual acuity, photophobia, and ocular pain^1^. The burden of DED can vary from mild discomfort to severe complaints that impact daily activities, reduce quality of life, and have significant socioeconomic implications^2^. The prevalence of DED increases with age and ranges from 5% to 50%^2^.

Accurate diagnosis of DED is complex and requires the application of a battery of tests, including questionnaires of patient-reported symptoms^3^, tear film break-up time (TFBUT)^4^, the Schirmer test^5^, ocular surface staining^6^, and meibomian gland functionality^7^. However, most of the tests lack consistency and reliability for diagnosing DED; therefore, they are subject to clinical interpretation based on experience^8^. The lack of a strong association between the signs and symptoms of DED is another challenge clinicians face in diagnosing and following-up patients with the disease^9^. Numerous ancillary diagnostic tests have been developed with the purpose of overcoming these challenges, including several patient-reported DED-specific questionnaires and new tools enabling the quantification of tear film characteristics. One of these tools is the measurement of tear osmolarity. Previous studies highlight tear hyperosmolarity as a significant pathophysiological factor in the development and the clinical course of DED^10–15^. Patients with DED present with elevated tear osmolarity compared to healthy controls^11,16^ and the hyperosmolarity increases with dry eye severity level (DESL)^16–18^.

*In vivo* measurement of tear osmolarity was not widely available for clinical practitioners until the TearLab osmometer (TearLab, San Diego, CA, USA) was approved by the US Food and Drug Administration in 2008. Since then, several studies have demonstrated that it is a reliable test with good performance for complementing DED diagnosis^16,19,20^*. In situ* analyses have also shown the inherent accuracy and precision of the TearLab osmometer^21^. Some studies have concluded that the TearLab osmometer is the best single marker for diagnosing and classifying DED levels of severity^11^ and for distinguishing DESL^18^. On the other hand, several clinical studies have raised questions on the diagnostic ability of the tear osmolarity measured with the TearLab osmometer in DED^13,22–24^. The varying results in the aforementioned studies might be due to the limitations of small sample size, studies conducted at multiple centers, and different diagnostic criteria for DED. Therefore, we conducted a single-center study with a large group of patients with DED (n = 757). Here, we explore whether tear osmolarity measurement with the TearLab system can be used as a diagnostic test along with clinical examination diagnostic methods for distinguishing patients with DED from healthy subjects.

## Methods

### Participants

This single-center, retrospective study was carried out at the Norwegian Dry Eye Clinic in Oslo, Norway. The study was conducted in accordance with the Declaration of Helsinki. The use of the data for the study from the Norwegian Dry Eye Clinic has been reviewed and approved by The Regional Committee for Medical & Health Research Ethics, Section C, South East Norway (REC). All participants gave a written informed consent for the data to be used for research purposes.

We included a total of **1514** eyes of **757** patients (mean age, 52.1 ± 16.1 years, range 8–89 years) diagnosed with symptomatic DED of different etiologies in at least one eye, with osmolarity measured using the TearLab osmometer in both eyes. The examinations took place from 2014 to 2018. The inclusion criteria were presence of clinically diagnosed DED of different severity levels (I–IV) according to the classification recommended by the 2007 Dry Eye Workshop (DEWS) developed by the Tear Film and Ocular Surface Society (TFOS)^25^ based on subjective feeling of ocular dryness (e.g. discomfort, pain), negative effect on visual ability (e.g. annoying or activity-limiting, OSDI > 12), conjunctival injection, conjunctival and corneal staining, signs of DED on cornea and tear film, presence of meibomian gland dysfunction, abnormal tear film break-up time and Schirmer test values. It is important to note that these signs and symptoms vary based on severity levels. For the control group, we recruited **29** healthy participants (**58** eyes). These participants were recruited from another ongoing study in collaboration with the Faculty of Dentistry, University of Oslo, Oslo (REK 2015/363). The participants were deemed healthy if the Ocular Surface Disease Index (OSDI)^3^ score was <12 and TFBUT ≥ 5 seconds. Control participants were excluded if they had any symptoms of DED (controlled with OSDI), seasonal allergy, used contact lenses or lubricating drops, had a history of refractive surgery, used of medications or had a systemic disease affecting tear production. Initially we had access to 65 control subjects who didn’t report symptoms of DED. However, clinical examinations revealed positive dry eye signs in some of them, and they were therefore excluded. We wanted to ascertain that controls had neither symptoms nor signs of DED. This rather strict inclusion criteria resulted in a smaller sample size of the control group.

### Dry eye examination

The participants underwent comprehensive dry eye assessment in the following order: subjective dry eye symptom evaluation using the OSDI questionnaire^3^ (Allergan, Irvine, CA, USA), tear osmolarity measurement using the TearLab osmometer^11^, inter-blink interval (IBI)^26^, TFBUT^4^, ocular surface staining (OSS)^6^, Schirmer I test^5^, meibum expressibility and meibum quality evaluation^7^, and meibography^27^. The examinations were carried out between 9 AM and 4 PM, and the participants were instructed not to use any eye drops within 2 hours prior to examination. Table 1 summarizes the ophthalmological work-up. All patients were examined between 9 am and 3 pm at the same clinic, where temperature and lighting were stable. Upon arrival to the appointment at the clinic, the patients were seated in a waiting area for at least 10 minutes. This was to ensure that the patients adjusted to indoor climate and did not have reflexive tearing after coming from outdoors. The tear osmolarity measurement was performed as described in the user manual^28^. The patient was seated with the chin tilted upwards and eyes directing towards the ceiling. The tip of the osmometer was positioned just above the lower lid in the lateral canthal area. The osmometer was gently lowered until the bottom of the tip touched the thin line of the tear film. Successful tear collection was confirmed by a beeping sound and disappearance of the green light. The osmometer was docked into the reader immediately (5–10 seconds), the test card code was selected, the OK button was pressed, and the test result was obtained. TFBUT was evaluated after instillation of 5 μl 2% fluorescein sodium, and an average of three readings was recorded. The ocular protection index (OPI) was calculated as the TFBUT divided by the IBI^29^. Ocular surface staining was graded according to the Oxford grading scheme^30^. Finally, meibomian gland expressibility and meibum quality were assessed by application of light pressure to the lower eyelids using a cotton swab^7^. Based on symptoms, ocular surface staining, TFBUT, Schirmer test, and meibomian gland functionality, the DESL in each eye was determined according to the TFOS DEWS dry eye severity grading system^25^.

**Table 1:** Summary of the ophthalmological work-up.

Examination	Scoring method	Pathological cut-off score
Tear osmolarity	Tear collection procedure was performed according to the user manual^28^. A nanoliter tear sample is obtained with a standard single-use micropipette, which is attached to the device and then transferred to a chip surface. A reading is obtained a few seconds after placing the test pen in the machine. Recommended threshold values in the literature indicating dry eye disease are ≥308 mOsm/L, intereye difference >8 mOsm/L and ≥316 mOsm/L	≥308 mOsm/L^a^, ≥316 mOsm/L, inter-eye difference>8 mOsm/L
TFBUT	The interval in seconds between the last complete blink and the first appearance of a dry spot, or disruption in the tear film following instillation of fluorescein	≤5 seconds
Inter-blink interval	Time between complete blinks in seconds	
OPI	The OPI is equal to the TFBUT divided by the IBI. The principle of the test is that when TFBUT < IBI, the ocular surface is at risk of focal dryness and consequent damage. If the OPI score is <1, then a patient’s cornea is at risk of exposure	<1
OSS	Following fluorescein instillation, the staining scores of the exposed cornea and interpalpebral conjunctiva are summarized using the Oxford grading scheme (range: 0–15)	>0
Schirmer I test	Paper test strips are placed in the lower lateral third of the conjunctival sac and the eyes are closed for 5 minutes, and wetting of the paper is measured in millimeters	≤10 mm/5 min
Meibum expressibility	Assessed in central five glands according to the number of expressible glands: 0 = all glands expressible; 1 = 3–4 glands expressible; 2 = 1–2 glands expressible; 3 = no glands expressible	>0
Meibum quality	Assessed in central eight glands scored on a scale of 0–3 for each gland: 0 = clear; 1 = cloudy; 2 = cloudy with debris (granular); 3 = thick, toothpaste-like, and the sum of the central eight glands is calculated (total score range, 0–24)	>0

### Statistics

Statistical analyses were performed with commercial software SPSS for Windows, version 23 (IBM, Chicago, IL, USA). The normal distribution of variables was verified by the Kolmogorov-Smirnov test. Intergroup analyses were carried out with Kruskal Wallis test. The Mann-Whitney U test was used to compare two groups. The ability of tear osmolarity measurement for predicting the pathological scores of other clinical dry eye tests was assessed using receiver operating characteristics (ROC) curve analysis. Pathological TFBUT, Schirmer I test, OSS, meibum expressibility, and meibum quality scores were defined as <5 sec, <10 mm/5 min, <1, >1, and >1, respectively. Values from the right and left eyes were analyzed separately. In addition, we calculated the average values from the right and left eyes. A p-value of <0.05 was considered statistically significant throughout the study. The analyses results are presented as the mean ± standard deviation (SD).

## Results

### Demographics

Among the patients, 629 (83.1%) were female and 128 (16.9%) (4.9:1) were male; the control group comprised 19 female (65.5%) and 10 male subjects (34.5%) (1.9:1). The mean age of the patient group was 52.2 ± 16.0 years; that of the control group was 44.9 ± 17.7 years (p = 0.201).

### Tear osmolarity in the patient group was not significantly different from the control group

Using the average values of both eyes the patient group showed significantly worse values for all clinical dry eye tests compared to the control group, except for tear osmolarity (Osm_Avg_), (310.6 ± 16.2 vs. 310.3 ± 19.4 mOsm/L, p = 0.754), (Table 2).

**Table 2:** Comparisons of clinical DED test results and osmolarity between patient and control group.

*Examination*	Patients	Controls	p-value
*OSDI*	27.1 ± 18.3	1.9 ± 2.2	<0.001
*Tear osmolarity (mOsm/L)*	310.6 ± 16.2	310.3 ± 19.4	**0.754**
*TFBUT (seconds)*	3.4 ± 3.2	8.6 ± 3.4	<0.001
*OPI*	1.5 ± 1.4	2.7 ± 1.4	<0.001
*OSS*	1.9 ± 1.9	0.6 ± 0.8	<0.001
*Schirmer I test (mm/5 min)*	14.3 ± 9.0	19.5 ± 10.7	<0.015
*Meibum expressibility*	1.8 ± 0.8	0.2 ± 0.5	<0.001
*Meibum quality*	8.9 ± 4.5	0.5 ± 1.1	<0.001

The patient group presented with worse subjective symptoms, shorter TFBUT, higher OSS, shorter Schirmer I test results and higher values of meibomian gland functionality. Apart from tear osmolarity, none of the mean test values in the control group reached pathological levels. In the patient group, the range of Osm_Avg_ was from 278.5 to 374.5 mOsm/L (95% confidence interval [95% CI] 309.5–311.8). Osmolarity levels in the right and left eye were 275–398 mOsm/L (95% CI 311.9–314.7) and 272–346 mOsm/L (95% CI 296–315.8), respectively. In the control group, the Osm_Avg_ ranged between 281.5 and 347.5 mOsm/L (95% confidence interval [95% CI] 301.9–318.7). Osmolarity levels in the right and left eyes were 281–369 mOsm/L (95% CI 304.7–323.7) and 275–398 mOsm/L (95% CI 306.6–309.3), respectively. Comparison of the parameters from the right and left eyes separately produced results that were consistent with those in Table 2.

### Tear osmolarity cut-off values could not distinguish DED participants from healthy participants

Analyses of the right eye revealed that 58% of the patients and 50% of the control subjects had osmolarity levels exceeding the suggested cut-off of 308 mOsm/L. The respective levels in the left eye were 42% and 39%. When the cut-off value of 316 mOsm/L was employed, 38% of right eyes and 27% of left eyes in the patient group had osmolarity levels exceeding the cut-off value. Similar results were obtained for the control group: 38% of the right eyes and 24% of the left eyes had osmolarity >316 mOsm/L. Whilst 62% of patients had inter-eye difference of >8 mOsm/L (range, 8–94) mOsm/L, the same was true for 74% of the controls (range, 8–66 mOsm/L).

Among the patients, about 10% had DESL 1. The vast majority of the patients had DESL 2 (72.5% of right eyes and 71.3% of left eyes), followed by DESL 3 in 14.4% of right eyes and 16.6% in left eyes. Only 1.3% of patients had DESL 4. We combined the participants based on DESL and created new groups: non-DED = DESL 0; mild DED = DESL 1 + DESL 2; severe DED = DESL 3 + DESL 4. Comparison of the groups based on DESL (Table 3) revealed that dry eye signs and symptoms were worsened in the mild DED group compared to the non-DED group and in the severe DED group compared to both the mild and the non DED group. However, there was not a significant difference (p = 0.123) in tear osmolarity values in the right eyes; non-DED 310.8 ± 21.9 mOsm/L, mild DED 312.7 ± 18.3 mOsm/L and severe DED 316.4 ± 23.5 mOsm/L. Similar to findings in the right eyes, subjective and objective parameters in the left eyes worsened as DESL increased. Severe DED group had tear osmolarity values of 312.9 ± 21.9 mOsm/L while mild DED had 306.9 ± 17.9 mOsm/L. The non-DED group showed tear osmolarity of 307.4 ± 21.1 mOsm/L.

**Table 3:** Comparison of dry eye parameters in non-DED, mild DED, and severe DED groups.

Examination	Right eye				Left eye			
	Non-DED (DESL 0)	Mild DED (DESL 1 + 2)	Severe DED (DESL 3 + 4)	p-value (ANOVA)	Non-DED (DESL 0)	Mild DED (DESL 1 + 2)	Severe DED (DESL 3 + 4)	p-value (ANOVA)
OSDI	2.2 ± 2.5	26.2 ± 17.4	31.4 ± 21.5	<0.001	2.2 ± 2.5	26.2 ± 17.4	31.4 ± 21.5	<0.001
Tear osmolarity (mOsm/L)	310.8 ± 21.9^a^	312.7 ± 18.3^b^	316.4 ± 23.5^c^	0.123	307.4 ± 21.1^a^	306.9 ± 17.9^d^	312.9 ± 21.9^c^	0.003
TFBUT (seconds)	8.0 ± 4.2	4.11 ± 3.5	2.3 ± 1.4	<0.001	8.45 ± 3.8	4.0 ± 3.5	2.3 ± 1.6	<0.001
OPI	2.5 ± 1.6	1.6 ± 1.5	0.9 ± 0.8	<0.001	2.9 ± 1.8	1.5 ± 1.5	0.9 ± 0.8	<0.001
OSS	0.68 ± 1.2	1.4 ± 1.5	4.4 ± 2.4	<0.001	1.1 ± 2.1	1.5 ± 1.6	4.6 ± 2.5	<0.001
Schirmer I test (mm/5 min)	19.3 ± 11.1	15.5 ± 9.8	9.9 ± 9.01	<0.001	19.6 ± 10.6	14.9 ± 9.3	9.4 ± 8.4	<0.001
Meibum expressibility	3.7 ± 1.4	1.7 ± 0.8	1.8 ± 0.8	<0.001	3.7 ± 1.6	1.8 ± 0.8	1.8 ± 0.9	<0.001
Meibum quality	1.27 ± 2.9	8.9 ± 4.6	7.7 ± 5.4	<0.001	1.0 ± 2.8	9.6 ± 4.7	7.9 ± 5.0	<0.001

^a^Difference between Non-DED and Mild DED, p > 0.05.

^b^Difference between Mild DED and Severe DED, p > 0.05.

^c^Difference between Non-DED and Severe DED, p > 0.05.

^d^Difference between Mild-DED and Severe DED, p = 0.002.

We attempted to determine whether tear osmolarity levels can discriminate DED pathological values employing ROC analyses. The results are shown in Table 4. The area under curve (AUC) was only significant for Schirmer I test in the right eyes (AUC = 0.544, p = 0.038) and left eyes (AUC = 0.546, p = 0.033). The other parameters did not demonstrate significant values. When using OsmAvg, statistically significant differences for the AUC values for average Schirmer I test (Avg Schirmer ≤ 10) and average ocular surface staining (Avg OSS > 3) were obtained. The remaining parameters did not show statistically significant differences.

**Table 4:** The results of ROC curve analysis for determining the optimum balanced sensitivity and specificity of tear osmolarity for discriminating DED pathological scores.

Variable	Average osmolarity (Osm_Avg_)		Variable	OD osmolarity^a^		Variable	OS osmolarity^b^	
	AUC^c^	p-value		AUC	p-value		AUC	p-value
OSDI	0.479	0.476	OSDI	0.483	0.582	OSDI	0.468	0.290
Avg TFBUT ≤ 5	0.516	0.522	OD TFBUT ≤ 5	0.512	0.658	OS TFBUT ≤ 5	0.500	0.986
Avg TFBUT ≤ 10	0.595	0.100	OD TFBUT ≤ 10	0.535	0.540	OS TFBUT ≤ 10	0.567	0.215
Avg OSS > 1	0.533	0.211	OD OSS > 1	0.536	0.111	OS OSS > 1	0.521	0.367
Avg OSS > 3	**0.556**	**0.019**	OD OSS > 3	0.552	0.062	OS OSS > 3	0.551	0.055
Avg Schirmer ≤ 5	0.554	0.068	OD Schirmer ≤ 5	0.546	0.089	OS Schirmer ≤ 5	0.548	0.070
Avg Schirmer ≤ 10	**0.544**	**0.043**	OD Schirmer ≤ 10	**0.544**	**0.038**	OS Schirmer ≤ 10	**0.546**	**0.033**
Avg ME > 1^d^	0,496	0.928	OD ME > 1	0.494	0.881	OS ME > 1	0.496	0.925
Avg MQ > 1^e^	0.460	0.478	OD MQ > 1	0.502	0.959	OS MQ > 1	0.462	0.476

^a^OD osmolarity – tear osmolarity levels in the right eye.

^b^OS osmolarity – tear osmolarity levels in the left eye.

^c^AUC – Area Under Curve.

^d^ME – Medibum expressibility.

^e^MQ – Meibum quality.

Moreover, we wanted to test whether there was a difference in osmolarity values between the two major forms of DED, aqueous deficiency dry eye (ADDE) and evaporative dry eye (EDE). In our dataset, 90.1% of the patients had a diagnosis of EDE due to MGD with various severity levels, while 9.9% of the patients were diagnosed with ADDE. Comparison of osmolarity levels between ADDE and EDE showed values of 326.8 ± 28.9 mOsm/L and 312 ± 18.2 mOsm/L (p = 0.123) in the right eye, respectively. Results for ADDE and EDE in the left eye were 304.5 ± 15.8 mOsm/L and 306.8 ± 17.5 mOsm/L, respectively, p = 0.809.

## Discussion

This retrospective study focused on investigating tear osmolarity measurement as compared to other subjective and clinical parameters of DED, including the scores of the OSDI, TFBUT, OSS, Schirmer I, and meibomian gland functionality tests. Our data show that osmolarity was not significantly different between the healthy controls and the patients (Table 2) and that a significant percentage of the healthy controls had tear osmolarity levels exceeding the recommended cut-off values (>308 mOsm/L, >316 mOsm/L, and inter-eye difference >8 mOsm/L). On the other hand, a substantial proportion of the patients with clinically diagnosed DED had tear osmolarity levels below the above cut-off values, suggesting a wide overlap of osmolarity between the controls and the patients. Using the mean value of the two eyes, osmolarity could be used to discriminate pathological Schirmer test values (<10 mm/5 min) (p = 0.043, optimum balanced sensitivity and specificity of 54% and 51%, respectively) and average OSS value (>3) (p = 0.019), but not any other clinical tests.

Tear film hyperosmolarity is believed to be an etiological factor in DED, hence the measurement of tear osmolarity has been considered important for diagnostic purposes^31^. Several measurement techniques have been used in the past^32,33^, with the Clifton and vapor pressure osmometers being the most commonly used methods. Despite high accuracy, sensitivity, and specificity^32,33^, these methods are not available for use at the point-of-care and require special setups that would need a significant amount of time. Clinicians did not have point-of-care diagnostic tests for measuring tear osmolarity until 2008, when the TearLab osmometer became commercially available. This technology uses a microchip, requiring only a 0.2-μl tear sample obtained without direct contact with the ocular surface. Lemp *et al*.^11^ concluded that tear osmolarity is the best single metric for diagnosing and classifying DED and suggested 308 mOsm/L as the most sensitive threshold between normal and mild DED. Similarly, Jacobi *et al*.^19^ proposed that a cut-off value of 316 mOsm/L demonstrated superior accuracy to other single tests for diagnosing DED. However, other studies demonstrated high variability of tear osmolarity in DED diagnosis^13,22–24^, and suggested that the TearLab osmolarity results should be interpreted with caution and in the context of other established methods. Accordingly, our data show that tear osmolarity levels measured with the TearLab osmometer were not significantly different between the controls and the patients.

If, as was done in the present study, osmolarity measured with the TearLab osmometer were used as the only diagnostic criteria for DED, the recommended cut-off value of 308 mOsm/L would exclude a significant proportion of patients (58% of right eyes and 42% of left eyes) with otherwise clinically diagnosed DED in our cohort. On the contrary, around 50% of the controls could have been diagnosed with DED when the same cut-off value was used. Using the cut-off value of 316 mOsm/L yielded similar results. Furthermore, another reference value commonly used for diagnosing DED, i.e., inter-eye difference >8 mOsm/L, failed to distinguish clinical DED from healthy eyes in the present study. Our data show that 62% of the patients and 74% of the controls had inter-eye difference >8 mOsm/L. These findings imply that tear osmolarity measurement with the TearLab osmometer has high variability and that it failed to distinguish patients with clinically diagnosed DED from healthy controls, which is in line with a recently published review by Baenninger *et al*.^24^. Our data differ from that of Lemp *et al*.^11^. The inconsistency might be caused by differences in the study population and DED diagnostic criteria. For example, the 314 subjects in their study were recruited from 10 centers, whereas we included 757 participants from a single center. In the present study, all clinical tests were performed by one experienced ophthalmologist with one or two assistants, which could have minimized the inter-observer variation in performing dry eye tests. The larger sample size might also have contributed to the statistical power of the analyses.

Despite the finding of Lemp and associates^11^, indicating a high accuracy of the TearLab osmometer in classifying DESL, the low discriminating ability of the TearLab osmometer and the overlap in osmolarity values have been reported previously^16^. In the present study, comparison of eyes with different DESL showed that in the right eye, all tests except the tear osmolarity measurement demonstrated inter-group differences. In the left eye, the severe DED group had the highest tear osmolarity level compared to the non-DED group (312.9 ± 21.9 vs. 307.4 ± 21.1, p = 0.467) and mild DED group (312.9 ± 21.9 vs. 306.9 ± 17.9, p = 0.002), but no statistically significant difference was detected between the non-DED and mild DED groups (307.4 ± 21.1 vs. 306.9 ± 17.9, p = 1.00) (Table 3). Comparison of DESL 0–4 as recommended in the DEWS guidelines yielded similar results.

There are several possible reasons for the lack of association between TearLab-measured osmolarity and the other clinical parameters. The osmolarity of the precorneal tear film differs significantly from that measured in the tear meniscus^13^, where the tears were collected by the TearLab system. The precorneal tear film in DED apparently has higher osmolarity levels, even spiking up to 800–900 mOsm/L in areas of tear film break-up^34^. Direct measurement of the precorneal tear film might show a stronger correlation with other clinical dry eye tests. Moreover, with the TearLab system, we probably measured the reflex tear, while earlier studies have suggested that pathological changes would be best obtained in basal tears rather than in reflex tears^13,35,36^. Using the TearLab osmometer might have led to reflex tear production, resulting in varying values.

### Strengths and limitations

The strength of the present study is the largest sample size in a published investigation of the utility of tear osmolarity measurement for diagnosing DED. Moreover, the data were collected at a single center. Well-trained ophthalmic assistant carried out standardized diagnostic examinations, and a senior ophthalmologist confirmed the examination results. One limitation of the present study is its retrospective design. The patients were not controlled for contact lens or systemic/ocular drug use, which might have affected tear osmolarity. Moreover, tear osmolarity levels were not measured repeatedly, thus it was not possible to detect the reliability or repeatability of the test. However, the patients were instructed not to use any eye drops two hours prior to their visit to the clinic, and the TearLab system was operated daily according to the manufacturer’s instructions before use. High prevalence of DED in age-matched population was another limiting factor in recruiting a large number of healthy controls with no DED signs and symptoms. Nevertheless, the number of control subjects was verified to be sufficient on statistical grounds. The study may appear to be underpowered due to a smaller sample size in the control group, as it is the smallest group is the one that mostly determines the study power. However, whether the study could possibly be under-powered also depends on the effect size. Since many highly significant results were found, it is evident that there are quite large effects in our data. Future prospective studies comparing a large number of DED-free controls are warranted.

In conclusion, tear osmolarity measured with the TearLab osmometer shows a large overlap between patients with DED and healthy individuals. Therefore, DED diagnosis relying on tear osmolarity measured with the TearLab osmometer can be inconclusive.
